# The British Journals: Pathology, Practical Medicine, and Therapeutics

**Published:** 1839-07

**Authors:** 


### PATHOLOGY, PRACTICAL MEDICINE, AND THERAPEUTICS.

### *Case of Ulceration of the Brain.* By G. P. May, M.D., Maldon.

Daniel Prior, aged 15, thrown from a horse, Jan. 17. On being visited two hours after the accident, was found to have received an extensive lacerated wound of the scalp, across the right parietal surface, by which the bone was denuded to a great extent. Considerable hemorrhage from the wound took place, amounting to more than a pint in quantity. He retained perfect possession of his senses, and complained little of his head, but referred his sufferings to his elbow-joint, which appeared to have received a violent contusion. On the fourth day after the injury he was able to come down stairs and exercise himself in the open air; by this period, a great part of the wound had healed by the first intention. For three weeks everything went on favorably, and the boy appeared to be the subject of little or no ailment. About the commencement of the fourth week symptoms of constitutional irritation began to manifest themselves; the pulse became quick, and the discharge assumed, for the first time, an unhealthy character; this condition continued, without much alteration, for six days, during which time he complained of pain of head.

Feb. 16th, he became comatose, in which state he continued until his death, which took place the following day.

*Sectio capitis.*—The wound of the head had an unhealthy aspect. Around the denuded portion of bone, between the scalp and periosteum, was an accumulation of pus, of a very offensive character; a small piece of the bone was carious, and exhibited some dark-coloured lamellæ when broken up by the handle of the scalpel; this condition did not extend through the external table. On raising the calvarium, two or three drachms of fetid pus escaped. The dura mater and subjacent membranes of the right hemisphere were completely eroded in two places, one about the size of a shilling, situated immediately posterior to the Sylvian fissure, the other of smaller dimensions, nearer the occipital region; the ulcers extended about three lines into the substance of the brain; their bases were hard, their edges ragged and coated with yellowish matter; the texture of the cerebral mass around the ulcers was apparently normal; a great quantity of lymph and pus was effused over the anterior lobe beneath the arachnoid membrane. The pia mater was highly injected, and the vessels of the convolutions and the sinuses were much distended with blood. The whole of the left hemisphere was very vascular; the corpus callosum, septum, and fornix, were in an advanced stage of softening; an excavation, the size of a pigeon's egg, was discovered in the posterior lobe, filled with fetid matter. The appearance of the cerebellum, left hemisphere, and ventricles, was natural. The thoracic and abdominal cavities were not examined.

This case is very interesting, as illustrative of the ambiguity which so frequently invests diseases of the encephalon. Every tissue here appeared to be the seat of some morbid action. The membranes bore evidence of acute and extensive inflammation. The cortical substance was, in two places, eroded to some depth; and the internal parts of the brain exhibited that alteration of structure most usually considered to be the result of inflammatory action; but until within twenty-four hours of death, there was scarcely a symptom diagnostic of any of these lesions. Up to this period the patient retained perfect possession of all his faculties, mental and physical; there was no delirium, paralysis, convulsion, or contraction of the limbs, which has been regarded by some French pathologists as indicative of cerebral ramollisement. These, with most of the conditions symptomatic of cerebral and meningeal inflammation, were altogether absent. Dr. Abercrombie, in his valuable treatise, has detailed some cases similar in this particular, and represents "the danger of being guided by system in our diagnosis of affections of the brain, and the necessity there still is for extensive and careful observation of facts in regard to this class of diseases." *Lancet. April* 13, 1839.

---

### *On the Bimeconate of Morphia, a new preparation of Opium.*
By Mr. P. Squire, Chemist to the Queen.

In reflecting on the artificial salts of opium, and the powers of opium itself, I

was led to suppose that the natural salts existing in this drug would be the best therapeutic agent, could it be separated from the other ingredients, which have often been found obnoxious to its soothing properties. I therefore instituted a series of experiments with a view to accomplish this task, and have obtained the *bimeconate of morphia* in a form which appears to have succeeded in a most satisfactory manner as the subjoined reports will show; and should the same success attend its employment generally by the profession, I shall feel a high gratification in having enhanced the value of a medicine which is so justly esteemed for its efficacy in relieving the sufferings of humanity.

*From* RODERICK MACLEOD, M.D.

I have now used the solution of the meconate of morphia in cases sufficiently numerous to enable me to form an opinion with respect to its powers.

It appears to me to be a very mild and efficient preparation, rarely producing headach or other discomfort. It has repeatedly answered in the most satisfactory manner where opium has disagreed, and has succeeded in some cases where the other salts of morphia (the acetate and hydrochlorate) had failed to give relief.

*From* A. T. THOMSON, M.D.

I have given a fair trial to your new preparation of opium, the bimeconate of morphia, which, being separated from many of the other constituents of opium, undoubtedly possesses anodyne properties superior to any of the salts of morphia in ordinary use. I have not had many opportunities of administering it in the hospital, but where it has been given in painful affections, its influence has been most striking.

I have administered it in three cases, in private practice, well calculated to illustrate its properties. The first was a neuralgic pain of the left side of the face, extending from the temple to the molar teeth of the upper jaw. It remitted during the day, but never wholly subsided, and returned with frightful severity at night. Among other means which were employed, anodynes of various kinds were tried, both topically and internally administered, namely, extract of opium in combination with calomel, hydrochlorate of morphia, acetate of morphia, Battley's sedative, and the black drop; belladonna in small and frequently-repeated doses, and in the form of plaster; hyoscyamus, both tincture and extract, and the extract of aconite. Temporary ease was obtained from the salts of morphia and the extract of aconite, but no permanent advantage resulted, although the pain assumed more of an intermittent character, and consequently disulphate of quina and arsenical solution had been freely administered. The strength of the patient was truly worn out for want of sleep, and under these circumstances your solution was prescribed. Thirty minims were prescribed in a fluid ounce and a half of camphor mixture, at bedtime, and two hours' sound sleep were procured, and on awaking, the patient felt the most satisfactory abatement of her sufferings. She was ordered to continue it in doses of eight minims, in combination with fifteen minims of the arsenical solution, every third hour during the day, and to repeat the full dose of thirty minims at bedtime, whilst, at the same time, the bowels were kept moderately lax. The most gratifying results followed this plan of proceeding; the pain gradually yielded; in less than a week she had comfortable nights, after which the dose of the bimeconate was rapidly diminished, its use discontinued during the day, and on the third week I had the satisfaction of leaving her perfectly free from the complaint.

The second case was one of wakefulness, without any apparent cause. All the usual preparations of opium had been tried without much benefit, and with suffering from headach and nausea on the mornings following the nights in which the narcotics were administered. The solution of bimeconate of morphia was given, in doses of twenty minims; it effectually procured sleep, and was not productive of the morning distress which had supervened the use of the other preparations of opium.

The third case was one of anomalous pain of the hip, extending down the thigh, which recurred three or four times in the twenty-four hours. The patient had been frequently attacked with rheumatism, and conceiving it to be connected with that disease, the part was cupped, and the guaiacum mixture, with small doses of blue pill, was prescribed. Little benefit resulted, until a week afterwards, when I ordered the part to be blistered, and the denuded surface to be dressed with a piece of lint dipped in your solution three times a day. The pain rapidly abated, and on the fourth day, it was completely gone.

From the limited experience which I have had of the use of the solution, I am of opinion that it possesses decided anodyne properties, and stimulates less than opium or its tincture, and is much more certain in its influence than any of the artificial salts, or other preparations of the drug.

*From* HENRY BRANDON, Esq.

I have much satisfaction in complying with your request to furnish you with an account of the effect which the meconate of morphia (same strength as laudanum) has had upon me, in comparison with the other preparations of opium that I have taken. My experience as an individual may probably be interesting, as well as somewhat useful. I have been the martyr to a spasmodic affection of the muscles for upwards of fourteen years, and was obliged, after trying every other means in vain, to have recourse to opium. I have taken crude opium, laudanum, aqueous extract of opium, black drop, liquor opii sedativus, and the acetate of morphia, and I must declare that not one of them has succeeded so well as the meconate of morphia, which relieves so much sooner, and without disturbing the stomach, leaving the system altogether in a more natural state of repose.

My experience has taught me that anodynes ought to be taken in solution, having found that when taken in the solid form they irritate by producing qualms in the stomach, frequently amounting to nausea. The aqueous extract does this less than the crude, but they are much more free from the exciting quality when in solution, and the solutions themselves, the purer and more free they are from the resin and other impurities, the less do they produce that unpleasant effect. Whether there be any additional cause why your meconate of morphia relieves so much quicker and strongly than others, you yourself best know.

My firm belief is that the qualms or unpleasant sensations in the stomach interfere materially with the soothing properties of the opium, and finding your preparation so free from these objections, added to the opium taste remaining a much shorter time on the palate than is the case with the other preparations of opium, I am induced to continue the use of it. The greatest quantity of opium that I ever took was in June, 1838, at which time I was suffering under a dreadful attack, when in seven consecutive days, I took as much as 540 grains of the aqueous extract of opium, which is equal to 810 grains of the crude.

I have taken ten grains of the acetate of morphia at a dose. My total consumption of opium for the year 1838 was equal to 49 pints 1 oz. of laudanum, and upwards. It is singular that none of the preparations of opium have ever confined my bowels or injured my appetite, although I take no exercise; indeed, a large dose of opium invariably increases my desire for food, and sometimes to a ravenous degree. *Lancet. April* 6, 1839.

---

*On Irritative or Pseudo-Fever, produced by the ingestion of warm fluids.* By NATHANIEL RUMSEY, M.D., of Beaconsfield.

MANY years ago I was myself the subject of typhus fever. Having passed through the disease, my debility was great, and convalescence interrupted by what appeared to be the daily occurrence of symptomatic fever; the cause not obvious, but suspected to be a pulmonary affection. After a great length of time, an unfavorable prognosis, and groundless anxiety in my friends, I recovered. The regular depression of bodily and mental power, which began about eleven o'clock A.M. daily, the heat of skin, quick pulse, and sweating, gradually left me. No confirmation was, by the termination, added to the opinion that local disease had existed; and the correct understanding of the case, to use the principle of the writer on education, was reserved for a future day. It became apparent to me at the distance of many years afterwards, made so by other facts, that these symptoms had been the effect of drinking daily for breakfast a large basin of warm milk, under the state of debility induced by the long-continued disease. I do not doubt that many similar facts may have occurred to me within the next few years, and as little understood; but in my subsequent experience, I have been brought to a confident belief that they are of no unfrequent occurrence, and that their cause is as clearly made out as it is important to be known.

These phenomena, which are, as it were, a mimicry of irritative fever, or that which is symptomatic of local disease, are particularly incident to patients reduced by loss of blood. I will therefore recite another case of recent occurrence, in a patient under that condition, to elucidate my meaning, and exhibit the importance of a correct view respecting it.

A lady having miscarried in the middle of the fourth month, suffered under repeated and alarming attacks of uterine hemorrhage for three weeks; in consequence, as it was supposed, and afterwards proved, of a retained placenta. I assisted in removing it at the end of that time, with much difficulty, and in separate portions, fearing much the consequences which might follow the unavoidable employment of the means used for this indispensable alternative. No symptoms but those of an exsanguined and weakened patient followed through the critical period of the next four days, after which, to the concern of my son, Mr. John Rumsey, who was in attendance, some circumstances arose which rendered him dissatisfied with the symptoms and progress of the patient. He reported that she had had, on several successive days, a paroxysm, marked by heat of skin, profuse sweating, great depression of all the powers of body and mind, a tongue thickly furred, slimy and dark in the centre, a pulse very feeble, with an increase of forty beats in a minute, and great despondency on her own part as to recovery. Hence, he expressed his fear that some source of irritation was still in existence (at that time a very probable thing), and augured unfavorably of the issue. Seeing her now, after some days' absence, a little enquiry led me to believe that this apparent fever was mere irritation, arising, as in the various other cases which I had seen, solely from receiving into the stomach a bulky, warm, and vapid fluid; and I was confirmed in this opinion by being able to trace, with the assistance of the patient, that the symptoms had, on one occasion, followed the use of two or three cups of tea, and at other times the liberal potation of warm chicken-broth. I was thus able, with great confidence and pleasure, to give a favorable prognosis.

I directed that evening that she should take a draught of good home-brewed ale, cold from the cask (Jan. 1 or 2), thus combining the exhibition of nourishment and a stimulus to the system with the application of cold to the stomach. The purpose was answered equal to my expectation. Animal food in small quantities and malt liquor were continued, and a careful disuse of the warm fluids observed. It is now several weeks since this change was made; no paroxysm has returned, and nothing has interrupted a daily improvement, the case having exhibited another instance of the peculiar affection I wish to describe.

I have seen this disturbance of the system usually assume the form of a paroxysm of fever, but on some occasions to consist of successive fits of fainting and hysteria; nevertheless coming on as a fit of fever, in point of time and duration.

If I might venture a conjecture as to the *modus operandi* of the warm fluids, I would suggest that the heart, morbidly sensible, responds too promptly to the stimulus of heat, when introduced into its neighbouring organ—the stomach, in so bulky a form as in the cases quoted and referred to; but in its endeavours to obey, incommoded by the pressure of bulk, and incompetent to a normal action by debility, makes its embarrassed struggles, disturbing the whole system by the failure and perversion of its function.

Nothing can be more simple than the indication of cure. Nourishment without bulk, stimulus regulated as to degree by judgment and discretion, not only without warmth, but actually cold internally; with rest, tonics, and attendance to warmth externally.

The importance of distinguishing the true character of these symptoms is not as the danger which they threaten, but as the danger which may be connected with an erroneous view; leading to a wrong prognosis, and the institution of remedies upon a wrong principle.

The previous debility or nervous temperament of the patient, the absence of satisfactory evidence of local disease, the effects of the concurrent use of warm fluids, and the febrile symptoms, often marked by unusual depression of spirits, may justly excite suspicions of the cause, and lead to a correct diagnosis; whilst nothing has served so powerfully to establish the view which I have taken as the subsidence of the symptoms upon the use of the cold tonic principle. The frequency of this condition has been such, that I fully believe its occurrence to the notice of any attentive observer having moderate opportunities could not be rare.

[The above communication is interesting and well worthy of attention; although we cannot consider the author's "conjecture as to the *modus operandi*" very satisfactory.] *Medical Gazette. May* 11, 1839.

---

*Proceedings of the Pathological Society of Dublin.*

1. *Pulmonary Calculus.* Mr. Crampton exhibited a mass of cretaceous matter, of stony hardness, taken from the lung of a phthisical patient; it was somewhat larger than a tennis-ball, and consisted of a series of spherical masses, which, when broken, presented the appearance of concentric laminæ; its composition was ascertained to be almost entirely carbonate of lime.

2. *Ileo-cæcal Abscess, with Perforation of the Intestine and Groin.*—Mr. Ferrall presented the recent parts in this case. The patient, a young girl, was admitted into the Meath Hospital, with tumour in the right iliac region, about fourteen days after the first attack; suppuration of the tumour had then occurred; the bursting of the abscess was soon indicated by a copious discharge of purulent matter from the bowels; soon after this another tumour formed in the upper part of the thigh, separated from the former by a deep sulcus corresponding to Poupart's ligament, below which an opening occurred, through which pus and ultimately fecal matter was discharged. Mr. Ferrall exhibited the mode of communication between the abscess and opening in the groin; the fistula took a direction at first downwards, and afterwards upwards and inwards, the omentum adhered to the parietes of the abdomen and cæcum; the communication from the abscess into the intestine was by two small openings separated by a slip of mucous membrane, and resembling the appearance often seen in the integuments when an abscess opens by a slough.

An important peculiarity in this case was the mode in which the matter had made its way externally, namely, by perforation of the iliac fascia, and descent on the outside of the femoral vessels.

Mr. Ferrall also showed that in this case the communication with the intestine did not, as Dr. Burne supposes, take place through the appendix vermiformis, the appendix being free from disease. The perforation had taken place from the abscess into the intestine, being the third form of the disease formerly described by Mr. Ferrall in the Edinburgh Journal.

3. *Separation and Discharge of the superior Epiphysis of the Os Femoris.* Dr. Carlisle exhibited the head of the femur, which had been separated from the shaft of the bone in consequence of scrofulous disease of the hip-joint. The patient, from whom the specimen was procured, was about twenty years of age, and of a scrofulous habit. At the age of fourteen he had suffered from acute rheumatism, which was followed by the usual symptoms of morbus coxarius. Suppuration took place in the joint, and matter escaped by several external openings. In this state he continued for four years; at the expiration of which period the head of the os femoris presented at one of the openings, and was extracted; its removal was followed by the rapid amendment of the boy's health. During the above period, several other bones became similarly diseased, and a large sequestrum was discharged from the humerus. The affected lower extremity was six inches shorter than the opposite limb.

4. *Disease of the Mitral Valve, without Valvular Murmur.* Dr. Stokes presented a specimen of disease of the mitral valve. It was principally remarkable on account of the physical phenomena observed during life. In this case the patient had long laboured under symptoms of morbus cordis with chronic bronchitis; and when the first was seen by Dr. Stokes, had a distinct and rough bruit de soufflet with the first sound of the heart. The second sound was unaffected. He remained under observation for several months, and always presented the same phenomena. In eighteen months he again came under Dr. Stokes's care, in a very advanced stage of dropsical disease. All bruit de soufflet had disappeared, and the heart merely presented the usual signs of hypertrophy. He died in about a month, during which time he was repeatedly examined, but no valvular murmur was ever detected; the heart was much enlarged; the left auricle greatly distended and thickened; the auriculo-ventricular valve was greatly diseased, and the orifice presented the semilunar contraction described by Mr. Adams; the opening was not more than two lines in breadth; numerous irregular deposits of earthy matter existed on the ventricular side of the valve. Dr. Stokes detailed the particulars of another case, in which a remarkable auricular contraction of the mitral valve had been found, in which no bruit de soufflet whatever had existed for a considerable time previous to death.

*Dublin Journal of Medical Science. March and May,* 1839.

---

*On the Effects of Colchicum and Lytta used externally.* By Thomas Laycock, Esq. House Surgeon of the York Hospital.

Some theoretical speculations led me to try the following liniment in rheumatism:—

℞ Tr. Rad. Colch.; Tr. Camph. aa. partes æquales. M.

The patient who used this was a tall groom (Richard Bould), under the care of Dr. Belcombe, subject to rheumatic attacks, and who at the time was unable to lift his arm, on account of rheumatism of the deltoid muscle. I was agreeably surprised to find that, after the third application, and within twelve hours after the first, he was able to raise his arm freely to his head. The relief was, however, only temporary, but the application was used with equal success so often as the pain recurred. The patient was subsequently attacked by small-pox (after vaccination), and nothing was heard of the rheumatic pains until he was convalescent, when they attacked his hip. He reminded me of the liniment, and one trial removed the pain. I now prescribed it for two or three out-patients, and these derived benefit. I then omitted the tincture of camphor, and I now find the groom is relieved with equal celerity and certainty by the tincture of colchicum-root alone. Relief so constantly follows its application in his case, that I cannot doubt its utility. When the loins are affected he cannot turn in bed unless the tincture be previously used. He rubs one or two tea-spoonsful on the part affected. I have found it equally successful in another case, in which the deltoid muscle was affected.

I believe it is well known that the tincture and powder of the melöe vesicatoria, or cantharis, is very useful in atony or paralysis of the bladder, especially of hysterical and aged people. I have found, however, that an emplastrum lyttæ applied to the loins is equally efficacious, and much more manageable. A female, confined to bed in the last stage of laryngeal phthisis, could not pass urine without raising herself upon her knees. She was at last too weak for the effort, and it became a question how the difficulty could be surmounted. I recommended an emplastrum lyttæ to be applied to the loins or sacrum, until she felt able to empty the bladder in the recumbent posture. In half an hour after the application she succeeded. She lived for three or four weeks subsequently, and the plaster was in almost daily use until she died. In most instances from one to two hours elapse before the desired effect is produced; in hysterical retention about the latter period. The plaster is useful in other cases. A man came to the hospital with a catheter in his bladder; he had not made water without it for three weeks. It was removed, and an emplastrum lyttæ applied to the sacrum for three or four hours; he never wanted the catheter again, and went away in a week quite well. I am not aware that this method of using the fly is mentioned by authors. *Med. Gaz. March* 16, 1839.

---

*On Rheumatism from Copaiba.* By A. B. Maddock, m.d., London.

[The following communication merits the attention of practitioners. The subject noticed in it demands and no doubt will obtain further investigation. There are few things in medicine more important than tracing *exactly* the causes of diseases.]

It is now some years since I expressed my belief that this complaint (rheumatism) generally originates from the administration of copaiba; and I take this opportunity of stating that further experience and observation have confirmed this opinion. I had, last February, under my care, a gentleman, aged 19, of a strumous habit of body, labouring under gonorrhœa, for which he was ordered, by a practitioner, a copaiba mixture, two days after taking which he was attacked by acute rheumatism in the knees and feet, which rendered him a complete cripple. He had been in this miserable state a fortnight, when he applied to me. I immediately ordered him to discontinue the copaiba, and substituted the iodide of potash and compound decoction of sarsaparilla; he was almost immediately relieved, and shortly cured. I will readily admit that copaiba is, in many cases, a most valuable medicine; but in persons of a scrofulous constitution I am convinced that it is a most pernicious excitant; and, if there be any disposition to rheumatism, will most certainly produce it. I am glad to find that my first communication attracted the attention of Dr. Sigmond, (vide *Lancet*, vol. ii., 1837-8, No. 24, p. 826,) and that his opinion coincides with my own. The potassæ iodidum is the medicine which I have employed, and have almost uniformly found it successful.

*Lancet. May* 25, 1839.

---

*Researches on the State of the Heart and the Use of Wine in Typhus Fever.* By WILLIAM STOKES, M.D., M.R.I.A., Honorary Fellow of the King and Queen's College of Physicians, &c.

[THIS is a paper of first-rate value, both in a pathological and therapeutic point of view; to which we call the attention of all our readers. The following extracts contain the more important observations, but the whole Essay ought to be read.]

If we compare the inexperienced man with him who has had a long-continued practice in fever, we may often observe that the former employs a too vigorous antiphlogistic treatment in the commencement of the disease, and delays the exhibition of stimulants until the powers of life are sunk too low, while the latter is much more cautious in husbanding the strength of his patient, and shows much less fear in resorting to wine and other stimulants. It is in determining on the use of wine in fever that the junior or inexperienced man feels the greatest difficulty; it is in its exhibition that he betrays the greatest uncertainty and fear.

The hospital physician will be frequently asked by students to state the principle on which he administers wine in fever. I conceive that the question may be thus answered. Typhus fever is a disease which has a tendency to a spontaneous and favorable termination, but one in the course of which the powers of life are attacked by a most malignant influence. By wine, food, and other stimulants, we support nature, until the struggle is past; so that, to use the words of an ancient author, which embody a more profound principle than appears at first sight, we "*cure the patient by preventing him from dying;*" that is to say, we prolong his existence until the natural and favorable termination of the disease arrives. We do not allow our patients to die of exhaustion; and bearing in mind the depressing influence they have to struggle with, we give stimulants at the proper time, and with a bold hand. We give our patients an artificial life, until the period arrives when nature and health resume their sway. Yet, though we may admire the practice of an experienced physician in the use of wine in fever, it will often be found that he has a difficulty in expressing any exact reason for adopting the practice in a particular case. His practice is founded on a knowledge which is often incommunicable, an almost instinctive perception of the necessity for stimulation, characteristic of the great physician, and only to be obtained by a long and close familiarity with the disease. But is there any rule by which *the inexperienced man* can be guided? any one distinct phenomenon, the observation of which is easy, and leading to an intelligible and communicable rule of practice?

Let us suppose a case of typhus on the tenth day of fever, and presenting severe symptoms of prostration, the pulse varying from 115 to 120. Wine is exhibited, and on the first day the pulse rises to 125, on the second to 130, and if on the third day there is no diminution, we may make a bad prognosis; and thus the following rule may be laid down, that when in a case where the symptoms seem to indicate wine, the pulse either does not come down, or increases in frequency under its influence, we may expect a bad result. These facts naturally lead to the examination of the state of the heart in typhus fever, and the cases in this report are so arranged as to exhibit together the condition of the heart, and the amount of wine employed. *In this investigation we have sought for an additional rule, drawn from the state of the heart itself, to guide the inexperienced man in the exhibition of wine,* and I am not without hopes, that in the careful study of the cardiac phenomena, an indication hitherto unobserved will be obtained. In typhus fever two opposite conditions of the heart may be observed; in the one the impulse becomes extremely feeble, or altogether wanting, while the sounds are greatly diminished in intensity; while in the other, the heart's action and sounds continue vigorous throughout the whole course of the disease. These opposite states are not necessarily revealed by the state of the pulse, or the warmth of the surface. We may observe a hot skin, while the action of the heart is almost imperceptible, and on the other hand a patient may be pulseless, cold, and livid for days together, while the heart is acting with the greatest vigour. The condition of the heart must be determined by the application of the hand and stethoscope to the infra-mammary and sternal regions.

[Then follow eighteen cases, minutely detailed, with the dissection of such as proved fatal.]

In these extracts [from Laennec and Louis] I have given all that has been discovered on the subject; no series of observations on the action of the heart in typhus fever has been published; I have commenced this enquiry, and have sought to derive some important indications of treatment from the existence of the phenomena now described. In the present state of the enquiry I wish it to be understood, that my observations are to be taken as referring principally to the epidemic of last year. Further researches must be made to establish how far they may be applicable to typhus in general; but I have little doubt, from studying the researches of Louis, and connecting the facts relative to the anatomical state of the heart, with those now observed as to its vital phenomena, that my observations will be found to have a very extensive application. The epidemic of last year was marked by all the signs of putridity. Dark-coloured and abundant petechiæ, sordes of the mouth, fœtor of the surface, extreme prostration, and stupor, were the prominent features of the disease; and in many cases bronchial and gastro-enteric irritation existed to a great degree. In many of the cases the bad symptoms were developed at an unusually early period, yet though recovery by crisis was by no means common, the convalescence was generally satisfactory, and the ultimate restoration to health complete. In several instances the disease was traceable to contagion. We may thus arrange the cardiac phenomena obtained in our typhus fever:—1. Impulse and sounds remaining unaltered; the action of the heart corresponding with that of the pulse. 2. Vigorous impulse, with distinct and proportionate sounds, with absence of pulse for many days. 3. Diminution of both sounds of the heart, with absence of great diminution of the impulse (fœtal character). 4. Diminution of the first sound; with cessation or great feebleness of the impulse. 5. Complete extinction of the first sound, the second remaining clear. 6. Predominance of the first sound, the second being extremely feeble. Of these the fourth and fifth were the most common. In the great majority of cases, however, the phenomena were as follow: I. Diminished impulse. II. Diminished first sound, particularly of the left cavities. With respect to the impulse we arrived at some unexpected results. In most cases, considered through the whole progress, the diminution and return of the first sound were accompanied with the diminution and return of the impulse. So far the phenomena were what we might expect. *But in some instances, at particular periods of the case, this accordance between the impulse and sound did not exist.* . . . It is difficult, or impossible, in the present stage of the enquiry, to offer any satisfactory explanation of these apparent anomalies; but it seems certain, that under the influence of the typhoid condition, the heart may have sufficient force to give an impulse with little or no sound, on the one hand; and on the other, its contractions may be accompanied by a sound, although the impulse be absent. Whether we are to explain these facts by referring to particular states of innervation of the heart, or to organic alteration in the muscular fibres, or their connecting cellular membrane, is still to be determined.

That the cause of the want of impulse, and feebleness or cessation of the first sound, is a softening of the heart, I have no doubt. The evidences in favour of this opinion may be thus stated:—I. That softening of the heart exists in typhus fever as a local disease, and without any analogous condition of the muscles of voluntary life. II. That in our dissections in the last epidemic, we meet with this softening of the heart, in cases which during life had presented the phenomena in question. III. That the physical signs indicate a debility of the left ventricle principally, and it is this position of the organ which is most often altered in consistence. IV. Laennec has stated, that in proportion to the severity of the putrescent phenomena, is the liability to softening of the heart. And the same observation is found to be true of the physical signs now described. If this softening of the heart be one of the secondary diseases of typhus, we should, as in the case of other lesions, observe something like periodicity in its phenomena. It should appear at a certain time, and decline after its proper period had expired. I have analyzed my cases with a view to these points, and the result is, that in most instances the signs of diminished impulse and first sound were developed at or about the sixth day, and the heart seemed again healthy at or about the fourteenth day. It is difficult to determine the period of the first development of the signs in many cases, as they existed on the admission of the patient, but still taking in these cases the dates of the disappearance of the signs, we get the following general results: Average date of appearance, sixth day. Average date of cessation, fourteenth day. One case has been excluded from this analysis; the patient was admitted on the tenth day, and the heart was not reported healthy till the twentieth. We thus get, as the duration of the phenomena, a period of about eight days. It is very probable, however, that the disease begins to be developed before the sixth, and that it subsides before the fourteenth day; for, as physical signs are our only means of detecting it, it is not likely that they would be well marked in its very first development, or indicate exactly the time of its subsidence.

I am decidedly of opinion that we cannot consider the softening of the heart in typhus as the result of carditis; it seems rather to be one of that class of affections not yet sufficiently examined, in which an infiltration of some peculiar substance takes place under the influence of the typhoid condition. This occurring in the heart seems to impair its functions to a great degree; but the rapid restoration of the heart to health points out that the disease has not materially impaired its organic condition. It is obvious that we can never meet with the affection in a very advanced condition, for death by syncope would occur after the contractility of the heart had been altered up to a certain point. Finally, I would draw the particular attention of my readers to the fact, that in the great majority of these cases the use of wine was followed by the happiest effects. I may safely refer to the cases in proof of this proposition; *and I believe that in the diminished impulse, and in the feebleness or extinction of the first sound, we have a new, direct, and important indication for the use of wine in typhus fever.* In some cases the existence of these phenomena at an early period of the disease, led us to anticipate the bad symptoms, and to commence in good time the use of the great remedy; and in others, notwithstanding the existence of severe visceral irritations, the use of stimulants has been adopted with the best success, from the same indication.

The following is the number of ounces of wine given in each of ten cases: 26, 36, 42, 60, 66, 88, 144, 156, 158, 170. "In no epidemic (says Dr. Stokes) did I ever before give so much wine. I never had such success in treatment."

The following are the conclusions deduced from Dr. Stokes from his investigations on this important subject: "1. That the condition of the heart in typhus fever must be determined by the application of the hand and stethoscope, the pulse being an uncertain guide. 2. That a diminished impulse, or a complete absence of impulse, occurs in certain cases of typhus fever. 3. That in such cases we may observe a diminished first sound, or even an absence of the first sound. 4. That both these characters may exist with a distinct pulse. 5. That although in most cases the diminution of the impulse and first sound coexists, yet that impulse may exist without corresponding first sound, and conversely, that the first sound may be heard although unaccompanied by impulse. 6. That these phenomena are most evident as connected with the left side of the heart. 7. That when the impulse and first sound are lessened or lost, the return to the healthy character is observed first over the right cavities. 8. That in some cases both sounds are equally diminished. 9. That in a few cases the first sound preponderates. 10. That these phenomena indicate a debilitated state of the heart. 11. That they may occur at an early period of the disease, and thus enable us accordingly to anticipate the symptoms of general debility. 12. That the existence of these phenomena, in a case of maculated adynamic fever, may be considered as pointing out a softened state of the heart. 13. That this softening of the heart seems to be one of the secondary local lesions of typhus. 14. That the diminution or cessation of impulse, the proportionate diminution of both sounds, or the preponderance of the second sound, are direct and nearly certain indications for the use of wine in fever.

*Dublin Journal of Medical Science. March*, 1839.

